# Effects of Influenza Strain Label on Worry and Behavioral Intentions

**DOI:** 10.3201/eid2308.170364

**Published:** 2017-08

**Authors:** Aaron M. Scherer, Megan Knaus, Brian J. Zikmund-Fisher, Enny Das, Angela Fagerlin

**Affiliations:** University of Iowa, Iowa City, Iowa, USA (A.M. Scherer);; University of Michigan, Ann Arbor, Michigan, USA (M. Knaus, B.J. Zikmund-Fisher);; Radboud University Nijmegen, Nijmegen, the Netherlands (E. Das);; University of Utah, Salt Lake City, Utah, USA (A. Fagerlin)

**Keywords:** Influenza, strain, worry, behavior, intentions, animal reservoir, hemagglutinin, neuraminidase, surface proteins

## Abstract

Persons who read information about a hypothetical influenza strain with scientific (H11N3 influenza) or exotic-sounding (Yarraman flu) name reported higher worry and vaccination intentions than did those who read about strains named after an animal reservoir (horse flu). These findings suggest that terms used for influenza in public communications can influence reactions.

Influenza strains are referred to in several ways by infectious disease experts, public health officials, clinicians, and the media when communicating with the public. These influenza strain labels can focus on where the strain originated (e.g., Spanish flu); the animal reservoir (e.g., avian/bird flu), or the hemagglutinin and neuraminidase surface proteins of the strain (e.g., H1N1 influenza). 

Changes in terms used to describe a health risk can shape responses to those risks (*1*–*5*). For example, using metaphors to describe influenza (e.g., the flu as an army invading the body) may increase influenza vaccination intentions of the public compared to literal descriptions (e.g., the flu is a virus infecting the body) (*1*). Labels could affect health behavior by the emotional responses they evoke (e.g., worry about infection) as a result of the terms used (*6*).

We tested how influenza labels affect vaccination intentions and worry about infection in a number of countries that have different cultures (*1*), vaccination policies (*2*), and experience with epidemics (*3**,**7*). After receiving exempt status from the University of Michigan Medical School institutional review board, we randomly recruited adults from a panel of internet users identified by using Survey Sampling International (SSI) (https://www.surveysampling.com/). Users were from 11 countries, the United States (n = 1,787) and 10 countries in different regions of Europe: northern [Finland (n = 1,554), Sweden (n = 1,539), Norway (n = 764)]; southern [Italy (n = 1,509), Spain (n = 1,604)]; eastern [Hungary (n = 998), Poland (n = 1,509)]; and western [Germany (n = 1,546), the Netherlands (n = 1,938), the United Kingdom (n = 1,762)]. We established quotas for age and gender to approximate the distribution of these characteristics in each country. Participants received modest compensation.

Respondents read a mock news article, ostensibly from an interview with a national health organization of the participant’s country, describing the spread of a pandemic influenza strain within their country (Technical Appendix). Each article contained information about the spread, symptoms, and severity of the virus and about the development of a vaccine. 

Each version of the article referred to the influenza strain by using 1 of 3 randomized labels: 1) “H11N3 influenza,” a surface protein label; 2) “horse flu,” an animal reservoir label; or 3) “Yarraman flu,” an exotic-sounding label (Yarraman is an Australian aboriginal term for “horse”). We used novel labels to avoid associations with and reactions to established influenza labels. The study included additional factors that were cross-randomized with the label factor and are not discussed here.

After reading the article, participants were asked to imagine that the described scenario was actually occurring and then rate the level of their worry about contracting influenza and plans to receive vaccination once a vaccine for this strain of influenza became available. Responses were on 7-point scales; higher values indicated greater worry or vaccination intentions. We tested for main effects of reactions to labels by using 1-way measured analysis of variance (ANOVA) with Bonferroni-adjusted planned contrasts. We used additional 2-way ANOVA tests to determine whether effects of the label manipulation differed across countries. We used the PROCESS macro for IBM SPSS Statistics 23 (IBM, Armonk, NY, USA) to conduct a mediation analysis and test for the effect of labels on vaccination intentions, controlling for worry.

Of 20,138 participants, 16,510 (82.0%) completed the full survey. The average participant age was 46.8 (range 18–99, SD 16.2) years; 49.8% were female.

Participants reported higher levels of worry about contracting the influenza strain when it was reported as “Yarraman flu” (mean 3.86, SD 1.83) or “H11N3 influenza” (mean 3.83, SD 1.82) compared with “horse flu” (mean 3.74, SD 1.86; F statistic [2–16,339] = 7.73, p<0.001). Participants also reported higher vaccination intentions when the strain was reported as “Yarraman flu” (mean 4.67, 1.99) or “H11N3 influenza” (mean 4.66, SD 2.03) compared with “horse flu” (mean 4.54, SD 2.04, F[10–16,339] = 6.48; p = 0.002). The effect of the influenza label on vaccination intentions was mediated by worry (Figure). Despite differences in reports of worry (F[10–16,339] = 100.07, p<0.001) and vaccination intentions (F[10–16,384] = 58.27, p<0.001) of participants in the 11 countries, the effects of the influenza label on worry (p = 0.281) and vaccination intentions (p = 0.467) did not significantly interact with country status.

**Figure:**
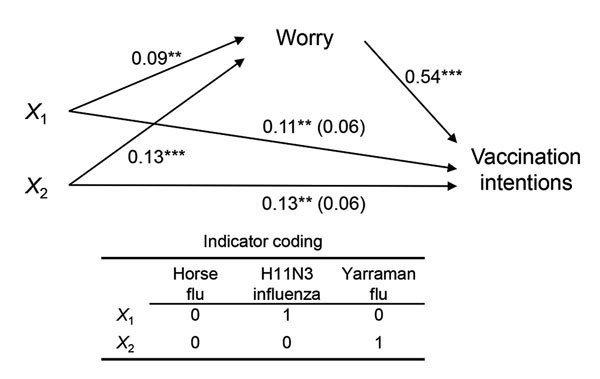
Regression coefficients for the effect of influenza labels on worry for infection and intentions for vaccination. Label conditions were dummy coded to estimate the effects of “H11N3 influenza” (X_1_) and “Yarraman flu” (X_2_) labels compared with the “horse flu” label. The effect of influenza labels on vaccination intentions, controlling for worry, is in parentheses. **p<0.01; ***p<0.001.

Our results indicate that the choice of disease labels for public communications about outbreaks cannot be made by personal preference. In this study, an animal reservoir label evoked weaker responses from participants than other labels. Although these results could be specific to the animal we chose, using an animal reservoir label may produce greater misconceptions (e.g., exposure to the animal necessary for transmission) that undermine suspicions of risk. Further research is needed to determine whether this effect is context-specific or generalizes to other animal reservoir labels for infectious diseases and whether our findings replicate in a nonhypothetical context.

Technical Appendix Description of 3 mock pandemic scenarios used in this study to compare whether labels, such as H11N3, Horse flu, or Yarraman flu, affected participants’ tendency to worry about infection with the virus and intentions to be vaccinated. The scenarios describe effects of fictional influenza viruses reported by fabricated public health personnel.
