# Supplementary material for: Effects of Influenza Strain Label on Worry and Behavioral Intentions
Source: Emerg Infect Dis. 2017 Aug;23(8):1425–6. doi: 10.3201/eid2308.170364 (PMC5547802; doi:10.3201/eid2308.170364)
Supplement: Technical Appendix — Description of 3 mock pandemic scenarios used in this study to compare whether labels, such as H11N3, Horse flu, or Yarraman flu, affected participants’ tendency to worry about infection with the virus and intentions to be vaccinated. The scenarios describe effects of fictional influenza viruses reported by fabricated public health personnel. [file 17-0364-Techapp-s1.pdf]

Article DOI: https://doi.org/10.3201/eid2308.170364

Effect of Influenza Subtype Label on Worry and Behavioral Intentions: A Multi-Country Experiment

Technical Appendix

Scenarios

In this study, participants read a mock news article describing the spread of a pandemic influenza within their country. To test how influenza labels affect worry about infection and vaccination intentions, participants received one of three different labels for the influenza strain described in the mock news article. Specifically, participants received a scenario which described the influenza strain by using either a surface proteins label (i.e., H11N3 influenza), an animal reservoir label (i.e., Horse flu), or an exotic-sounding label (i.e., Yarraman flu). Example scenarios that participants in the United States received are included below. For the purpose of this appendix, labels are in bold text, but appeared in normal font for study participants.

Scenario 1: Use of surface protein label - H11N3 Influenza

CDC Reports H11N3 Influenza Infecting the US

The **H11N3 Influenza** has been infecting the US. The number of people reported to have **H11N3 influenza** has risen recently according to health officials at the Centers for Disease Control and Prevention (CDC).

Health officials are confident that this outbreak will be a bad one. "**H11N3 Influenza** is a virus quickly infecting the US," says Dr. Alexander Dewitt, the lead expert with the CDC. "We are seeing it progress and move from city to city with alarming speed."

"**H11N3 Influenza** is a severe virus, and people are at risk for serious illness or death," said Dr. Dewitt. "Although we believe that many people will only have relatively mild to moderate symptoms, we expect to see some severe cases, some of which will lead to death."

Most of those who have gotten sick have experienced moderate fever with cough and body aches. Symptoms generally go away without medicine. Some extreme cases have required patients seeing a doctor and 1–2 days of hospitalization. These individuals experienced difficulty breathing, sudden dizziness, and severe persistent coughing.

Dr. Dewitt emphasized that the estimates of the symptoms that those with **H11N3 Influenza** will experience are based on the information currently available to health officials.

With a growing number of cases of people getting the virus, Dr. Dewitt promised that the soon to be released vaccine will prevent people from getting **H11N3 Influenza**. Vaccines stop the spread of diseases by using the body's natural response to prevent us from getting sick. Specifically, the **H11N3 Influenza** vaccine will create antibodies, the proteins of the body that identify a virus so the immune system can respond more quickly to **H11N3 Influenza** when it is encountered again.

Dr. Dewitt assured that the vaccine will be safe, effective, and is being tested extensively. "The **H11N3 Influenza** vaccine uses many of the same elements of vaccines from previous flu seasons and is undergoing standard development and testing. We have every reason to believe the vaccine will be effective, and it's the best option available right now to protect people against the **H11N3 Influenza** virus," said Dr. Dewitt.

"The vaccine is the most effective way we have to prevent the growth of **H11N3 Influenza**," he said.

Once the vaccine becomes available, Dr. Dewitt urged people to get vaccinated, even if they have questions about their risks of **H11N3 Influenza** or the effectiveness of the vaccine.

Scenario 2: Use of animal reservoir label – Horse flu

CDC Reports Horse Flu Infecting the US

The **Horse flu** has been infecting the U.S. The number of people reported to have **Horse flu** has risen recently according to health officials at the Centers for Disease Control and Prevention (CDC).

Health officials are confident that this outbreak will be a bad one. "**Horse flu** is a virus quickly infecting the US," says Dr. Alexander Dewitt, the lead expert with the CDC. "We are seeing it progress and move from city to city with alarming speed."

"**Horse flu** is a severe virus, and people are at risk for serious illness or death," said Dr. Dewitt. "Although we believe that many people will only have relatively mild to moderate symptoms, we expect to see some severe cases, some of which will lead to death."

Most of those who have gotten sick have experienced moderate fever with cough and body aches. Symptoms generally go away without medicine. Some extreme cases have required patients seeing a doctor and 1–2 days of hospitalization. These individuals experienced difficulty breathing, sudden dizziness, and severe persistent coughing.

Dr. Dewitt emphasized that the estimates of the symptoms that those with **Horse flu** will experience are based on the information currently available to health officials.

With a growing number of cases of people getting the virus, Dr. Dewitt promised that the soon to be released vaccine will prevent people from getting **Horse flu**. Vaccines stop the spread of diseases by using the body's natural response to prevent us from getting sick. Specifically, the **Horse flu** vaccine will create antibodies, the proteins of the body that identify a virus so the immune system can respond more quickly to **Horse flu** when it is encountered again.

Dr. Dewitt assured that the vaccine will be safe and effective. "The **Horse flu** vaccine uses many of the same elements of vaccines from previous flu seasons and is undergoing standard development and testing. We have every reason to believe the vaccine will be effective, and it's the best option available right now to protect people against the **Horse flu** virus," said Dr. Dewitt.

"The vaccine is the most effective way we have to prevent the growth of **Horse flu**," he said.

Once the vaccine becomes available, Dr. Dewitt urged people to get vaccinated, even if they have questions about their risks of **Horse flu** or the effectiveness of the vaccine.

Scenario 3: Use of exotic-sounding label – Yarraman flu

CDC Reports Yarraman Flu Infecting the US

The **Yarraman flu** has been infecting the US. The number of people reported to have **Yarraman flu** has risen recently according to health officials at the Centers for Disease Control and Prevention (CDC).

Health officials are confident that this outbreak will be a bad one. "**Yarraman flu** is a virus quickly infecting the US," says Dr. Alexander Dewitt, the lead expert with the CDC. "We are seeing it progress and move from city to city with alarming speed."

"**Yarraman flu** is a severe virus, and people are at risk for serious illness or death," said Dr. Dewitt. "Although we believe that many people will only have relatively mild to moderate symptoms, we expect to see some severe cases, some of which will lead to death."

Most of those who have gotten sick have experienced moderate fever with cough and body aches. Symptoms generally go away without medicine. Some extreme cases have required patients seeing a doctor and 1–2 days of hospitalization. These individuals experienced difficulty breathing, sudden dizziness, and severe persistent coughing.

Dr. Dewitt emphasized that the estimates of the symptoms that those with **Yarraman flu** will experience are based on the information currently available to health officials.

With a growing number of cases of people getting the virus, Dr. Dewitt promised that the soon to be released vaccine will prevent people from getting **Yarraman flu**. Vaccines stop the spread of diseases by using the body's natural response to prevent us from getting sick. Specifically, the **Yarraman flu** vaccine will create antibodies, the proteins of the body that identify a virus so the immune system can respond more quickly to **Yarraman flu** when it is encountered again.

Dr. Dewitt assured that the vaccine will be safe and effective. "The **Yarraman flu** vaccine uses many of the same elements of vaccines from previous flu seasons and is undergoing standard development and testing. We have every reason to believe the vaccine will be effective, and it's the best option available right now to protect people against the **Yarraman flu** virus," said Dr. Dewitt.

"The vaccine is the most effective way we have to prevent the growth of **Yarraman flu**," he said.

Once the vaccine becomes available, Dr. Dewitt urges people to be vaccinated, even if they have questions about their risks of **Yarraman flu** or the effectiveness of the vaccine.
